# Patient-Provider Discussions on Lung Cancer Screening: Insights From the Health Information National Trends Survey (HINTS)

**DOI:** 10.7759/cureus.106943

**Published:** 2026-04-13

**Authors:** Mohamad El Labban, Felix Wireko, Anaelle Zimmowitch, Yewande Odeyemi, Wigdan Farah

**Affiliations:** 1 Internal Medicine, Mayo Clinic Health System, Mankato, USA; 2 Division of Pulmonary and Critical Care Medicine, Mayo Clinic, Rochester, USA

**Keywords:** chronic lung disease, hints, lung cancer screening, patient-provider communication, preventive care

## Abstract

Background

Lung cancer risk is significantly elevated in individuals with chronic lung diseases (CLDs), including asthma, emphysema, and chronic bronchitis. Despite this, lung cancer screening (LCS) remains underutilized in this high-risk population. This study aimed to examine the factors associated with patient-provider discussions about LCS among adults with CLD.

Methodology

Using data from four cycles of the Health Information National Trends Survey (HINTS) from 2013 to 2022, we examined patient-provider communication patterns about LCS among respondents over the age of 50 years with a smoking history and CLD.

Results

Of the 828 eligible respondents, only 26.3% reported having discussed LCS with a healthcare provider. Older age, non-Hispanic ethnicity, and prior discussions about colorectal cancer screening were associated with higher rates of LCS conversations, while food insecurity significantly decreased the likelihood of such discussions. Notably, conversations peaked in 2020 but declined sharply by 2022, potentially reflecting disruptions in preventive care due to the COVID-19 pandemic.

Conclusions

Our findings highlight the persistent communication gap around LCS in CLD patients, emphasizing the need for improved provider engagement and awareness. Socioeconomic disparities and limited preventive care access continue to influence screening uptake. Interventions that promote shared decision-making, address social determinants of health, and integrate LCS discussions into routine care could enhance early detection and reduce lung cancer-related morbidity and mortality in this vulnerable population.

## Introduction

Chronic lung diseases (CLDs) and lung cancer are among the leading causes of morbidity and mortality worldwide and in the United States, with historically underserved populations and individuals from rural communities bearing a disproportionate burden of the disease [1-5]. The chronic inflammation, oxidative stress, and tissue remodeling seen in CLDs create a conducive environment for carcinogenesis, leading to a higher incidence of lung cancer in CLD patients compared to the general population, even after adjusting for smoking and other risk factors. Current studies demonstrate that individuals with CLDs, including chronic obstructive pulmonary disease (COPD), chronic bronchitis, and emphysema, are at increased risk of developing lung cancer when compared to the general population, with reported prevalence ranging between 3% and 9% [6,7]. Notably, even non-smokers with COPD have a significantly higher incidence of lung cancer than those without COPD, reinforcing COPD as an independent risk factor beyond smoking [8].

Over the last couple of years, smoking cessation interventions, along with early detection and timely high-quality treatment, have remained the primary recommended measures in improving lung cancer outcomes [9-12]. There are reported benefits of early detection and efficacy of low-dose computed tomography (LDCT) in improving prognosis and reducing mortality rates for high-risk smokers. Despite this, lung cancer screening (LCS) rates across the United States have unfortunately remained alarmingly low, particularly in the context of other more successful cancer screening programs [13,14].

The complex interplay between social, behavioral, and healthcare delivery access factors contributes to the current challenges hindering the uptake and adherence to lung cancer screening. Although researchers have yet to fully understand the breadth of these interactions, several studies have highlighted multiple barriers to screening at the individual and system levels. Several major themes have emerged, including fear of lung cancer, screening risks, knowledge deficits, and misconceptions about screening benefits, as well as demographic factors [15-17]. While individual-level barriers play a significant role in lung cancer outcomes, it has become increasingly clear that factors outside of the individual level are instrumental to the current low uptake of LCS.

Over the years, effective patient-provider communication has emerged as a key component in improving cancer screening adherence, with studies demonstrating a strong association between provider recommendations, shared decision-making, and increased screening rates [18]. A 2023 study found that patient-provider communication using this approach led to a 57.6% increase in LCS uptake [19]. The National Cancer Institute’s Health Information National Trends Survey (HINTS) assesses knowledge, access, attitudes, and cancer-related information use among U.S. adults [20]. While previous studies using HINTS have consistently reported low rates of patient-provider discussions on LCS and identified various influencing factors, data on patients with CLDs, who are at higher risk for lung cancer, remain scarce [21,22], underscoring the need for further research. To address this, our study aimed to explore the pattern of the patient-provider communication regarding LCS in individuals with CLDs. By focusing on this high-risk group, we seek to generate insights that could improve screening rates and clinical outcomes for CLD patients. Of note, our group previously presented related material as an abstract at the AORTIC 2025 Conference on Cancer in Africa, held in Hammamet, Tunisia.

## Materials and methods

**Figure 1:**
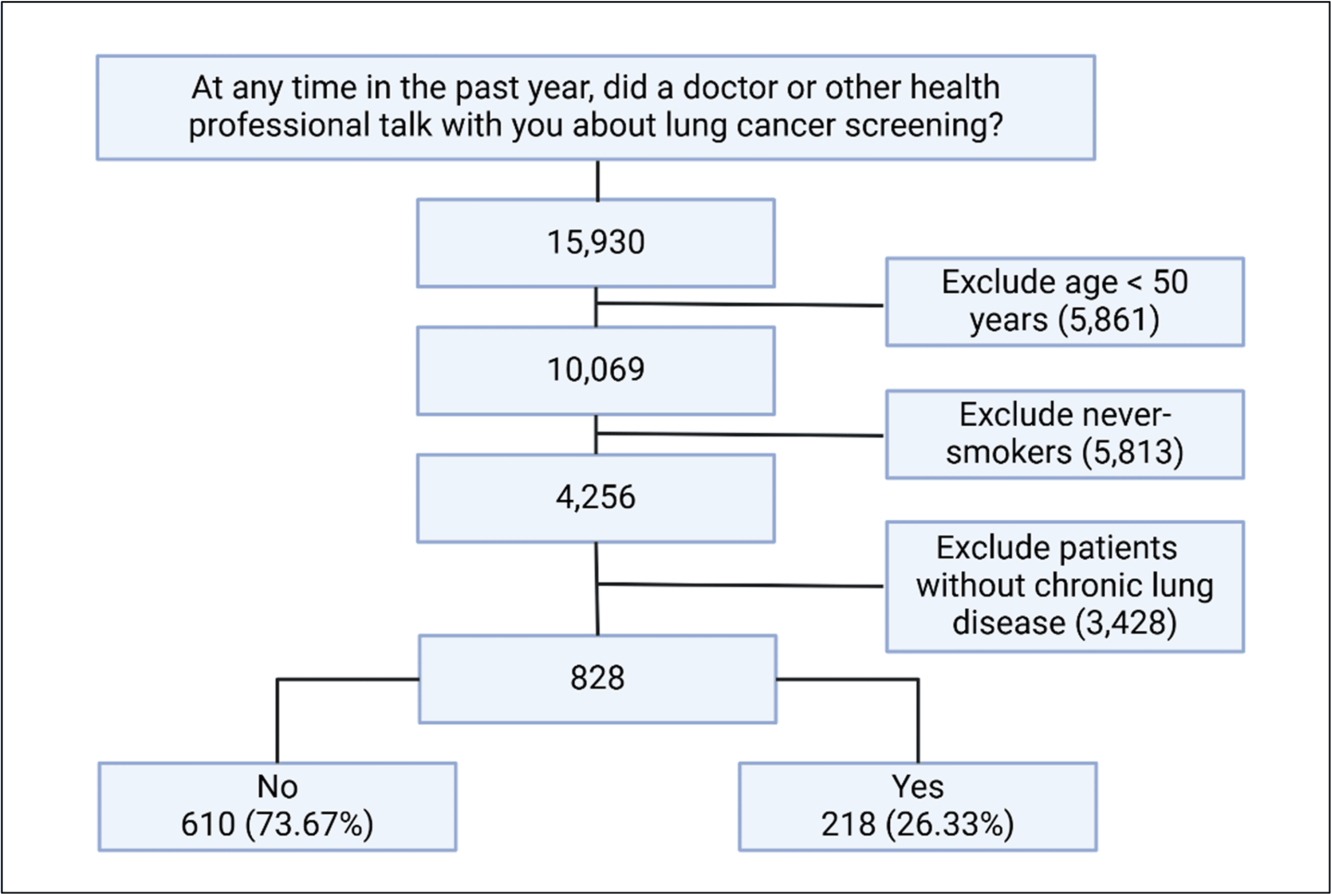
Flow diagram illustrating respondent selection.

We analyzed data from four national surveys (HINTS 4 Cycle 3-4 (2013-2014), HINTS 5 Cycle 1-4 (2017-2020), and HINTS 6 (2022)). We included respondents aged >50 years with a smoking history, aligning with the 2021 United States Preventive Services Task Force (USPSTF) LCS criteria, with a focus on patients with CLDs (asthma, emphysema, or chronic bronchitis) (Figure 1) [11]. This study did not use any private identifiable information and thus did not require institutional review board oversight.

Patient-provider discussion about LCS was assessed with the following questions: “At any time in the past year, did a doctor or other health professional talk with you about having a low-dose CT (LDCT) scan to check for lung cancer?.” and “At any time in the past year, have you talked with your doctor or other health professional about having a test to check for lung cancer?.” Responses were categorized as “yes” and “no.” We obtained information about respondents’ demographics, including age, gender, race, ethnicity, marital status, education, employment status, annual household income, insurance, and health literacy.

All data were analyzed using survey weights based on population estimates to account for non‐response and coverage error, making the results more generalizable to the population based on HINTS protocols. As is best practice with the HINTS data, jackknife replicate weights were used to provide bias‐corrected variance estimates [23]. Descriptive statistics were used to summarize the sample’s sociodemographic and clinical characteristics. Standard errors were estimated using the Wald method to compare the two cohorts. Race/ethnicity, socioeconomic status (income, education), and smoking status were selected as theory-driven covariates based on their established roles in shaping lung cancer risk, screening eligibility, and access to care. The USPSTF and recent cohort studies have shown that these factors are independently associated with disparities in LCS discussions and uptake [10,24,25]. To assess model robustness, we first evaluated the presence of multicollinearity among predictor variables using variance inflation factors (VIFs). A linear regression model including all independent variables was run before logistic regression, and the mean VIF was 1.08, indicating no concerning multicollinearity. Additionally, we assessed the overall goodness of fit of the final survey-weighted logistic regression model using a design-based Pearson goodness-of-fit test, which accounts for complex survey design and returns an F-statistic. A non-significant result (Prob > F > 0.05) suggests adequate model fit. The test yielded an F(9, 335) of 0.62 with a p-value of 0.78, indicating no evidence of poor model fit due to overfitting or other sources of misfit. All data analysis was conducted using Stata/BE 17.0 (StataCorp., College Station, TX, USA). Statistical significance was defined as a p-value <0.05.

## Results

A total of 828 respondents with CLDs were included in the analysis, of whom 218 (26.33%) discussed LCS with their healthcare provider (Figure 1). Respondents who reported discussing LCS with their provider were older (69 vs. 66 years, p < 0.01), with a higher proportion of males (58.2% vs. 57, p = 0.09), and from a non-Hispanic ethnic background (97% vs. 92%, p = 0.033) compared to those who did not.

Individuals who had previously discussed colorectal cancer screening with a provider had higher rates of LCS discussion (88.4% vs. 66.9%, p = 0.001). Current smokers reported LCS discussions more often than former smokers (42% vs. 33%), though the difference was not statistically significant (p = 0.12). Additionally, individuals experiencing food insecurity had lower rates of LCS discussion (11.2% vs. 34.7%, p = 0.01). Discussions about LCS peaked in 2020 at 37.29%, followed by a decline to 19.32% in 2022. No significant differences were observed based on smoking status, insurance status, personal history of cancer, or other sociodemographic and healthcare access factors (Table 1). In the multivariate analysis, prior discussion of colorectal cancer screening was significantly associated with a higher likelihood of discussing LCS (odds ratio (OR) = 3.24, 95% confidence interval (CI) = 1.13-9.2, p=0.028), while food insecurity was associated with lower odds of such discussions (OR = 0.19, 95% CI = 0.05-0.76, p = 0.019).

**Table 1:** Demographic characteristics and factors associated with discussions on lung cancer screening by response group. *: How confident are you in filing medical forms by yourself? High health literacy was defined as those who answered “Very” and “Somewhat.” Low health literacy was defined as those who answered “A little” and “Not at all.” **: P-value <0.05 is considered statistically significant.

Characteristics unweighted no (weighted %)	At any time in the past year, did a doctor or other health professional talk with you about lung cancer screening?		P-value
	No	Yes	
Median age, interquartile range (25%–75%) (years)	66 (59–74)	69 (63–75)	<0.01**
Year			0.11
2014	130 (23.38)	33 (17)	-
2017	115 (28.5)	44 (26.37)	
2020	137 (22.31)	63 (37.29)	
2022	228 (25.79)	78 (19.32)	
Female	354 (53)	114 (41.8)	0.09
Education			0.9
Less than high school	84 (18.76)	35 (19.7)	-
High school graduate	142 (27.3)	59 (28.16)	
Some college	229 (40)	77 (4024)	
College graduate	152 (14)	47 (11.88)	
Race			0.25
White	448 (86.36)	156 (78.75)	-
Black	92 (7.6)	40 (11.81)	
Asian/Pacific Islander	11 (2.58)	5 (2.57)	
Other	30 (3.45)	15 (6.85)	-
Ethnicity			0.033**
Non-Hispanic	514 (92)	182 (97)	-
Hispanic	42 (8)	11 (3)	
Insurance			0.0503
Uninsured	21 (4)	4 (0.9)	
Insured	359 (96)	134 (99.1)	
Marital status			0.45
Married or living with a partner	250 (59)	81 (55)	-
Single, divorced, separated, or widowed	355 (41)	133 (45)	
Occupation			0.42
Employed	138 (33.4)	54 (29)	-
Unemployed, retired, disabled, other	457 (66.6)	156 (71)	
Income less than 50,000 US$	385 (57)	147 (64)	0.23
Health literacy*			0.48
Low health literacy	63 (10.41)	16 (8)	-
High health literacy	545 (89.59)	202 (92)	
Smoking status			0.12
Current smoker	207 (33)	97 (42)	-
Former smoker	403 (67)	121 (58)	
General health			0.7
Fair and poor	267 (44.8)	109 (47.1)	-
Good to excellent	335 (53.2)	108 (52.9)	
Family history of cancer	441 (75.9)	166 (80.5)	0.37
Past medical history			-
Personal history of cancer	169 (20)	87 (29.5)	0.07
Diabetes mellitus	222 (36.2)	73 (32.82)	0.58
High blood pressure	413 (66.6)	160 (72.9)	0.27
Heart disease	160 (25.9)	62 (31.6)	0.28
Depression	263 (39.28)	89 (39.72)	0.94
Access to the online portal in the last 12 months	202 (39.5)	87 (40.63)	0.86
Number of doctor appointments in the last 12 months			0.38
1-4 visits	325 (60)	104 (54)	-
More than four visits	238 (40)	101 (46)	
Talked about colorectal cancer screening with a healthcare professional	148 (66.9)	63 (88.4)	0.001**
Food insecurity	64 (34.7)	14 (11.2)	0.01**
Transportation insecurity	43 (25)	15 (20)	0.62
Housing insecurity	38 (21)	13 (19.4)	0.87

## Discussion

In this retrospective study, we conducted a secondary analysis of HINTS data to examine patient-provider communication regarding LCS among high-risk patients with CLDs. Our findings reveal that only 26.3% of at-risk respondents reported discussing LCS with their healthcare provider. Notably, prior discussions about other preventive measures, such as colorectal cancer screening, were associated with increased likelihood of LCS discussions, whereas gender, race, cancer history, and health insurance did not have a significant impact.

Despite established guidelines, LCS rates in the United States remain low. Data from the 2022 Behavioral Risk Factor Surveillance System (BRFSS) show that only 18.1% of eligible individuals were up to date with LCS, with significant variation across states (9.7% to 31.0%) [26]. In 2018, these rates were even lower (5%), in comparison to the average 60-70% reported screening rates for colon and breast cancer by the National Cancer Institute [5,27]. Previous studies have demonstrated that patient-provider discussion is a key tool to improve screening uptake [18,28]. In our study, prior discussions on colorectal cancer screening were associated with increased LCS discussions (OR = 3.31, 95% CI = 1.46-7.4, p = 0.004), aligning with prior research [22]. This correlation may suggest that prior colorectal cancer screening discussion may serve as an entry point for broader preventive health measures, including LCS [29]. This highlights the potential value of health policies that focus on leveraging preventive discussions as a catalyst for broader engagement, in addition to addressing the role of combined screening strategies to help with improving LCS outcomes, which align with previously published data from modeling studies demonstrating improvement in outcomes associated with joint screening strategies.

Social determinants of health significantly influence LCS discussions and outcomes. In our analysis, food insecurity was associated with a lower likelihood of LCS discussions (OR = 0.27, 95% CI = 0.09-0.81, p = 0.02), reinforcing the role of social determinants of health in preventive care engagement. This finding aligns with prior studies showing an association between food insecurity and non-adherence to cancer screening [30]. While financial instability can lead individuals to prioritize immediate needs over preventive healthcare, our results also highlight a pattern of lower provider engagement in preventive care services with patients from more vulnerable backgrounds, highlighting the need for targeted interventions to support these underserved populations.

Interestingly, compared to the previous years, there was a dramatic decline in LCS discussions after 2020 (from 37.3% to 19.3%), potentially implicating the COVID-19 pandemic as a catalyst in the disruption of preventive healthcare [26]. Similarly, Oakes et al. noted that breast, cervical, and colorectal cancer screening rates declined by 40%, 36%, and 45%, respectively, during the COVID-19 pandemic [31]. However, further studies are needed to examine this effect and whether any improvement has been noted post-pandemic.

Our study found that non-Hispanic respondents were more likely to have LCS discussions compared to Hispanic respondents (26.1% vs. 20.1%, p = 0.033), though other studies have found no significant differences [21,32]. Huo et al. (2019) identified both non-Hispanic Black and Hispanic populations as more likely to discuss LCS. Additionally, previous malignancy and insurance status were associated with increased LCS discussions in other studies, contrary to our findings. Interestingly, in our study, smoking status did not significantly influence LCS discussions, despite its well-documented association with lung cancer risk [8]. Prior studies additionally suggest that smoking history increases the likelihood of LCS discussions [22,33]; however, we observed that while current smokers reported discussions more frequently than former smokers (42% vs. 33%), this difference was not statistically significant (p = 0.12). This suggests that although smoking history is a key criterion for LCS eligibility, it may not be adequately emphasized in clinical conversations. Given that even never-smokers with COPD have an elevated lung cancer risk [8], there is a need for broader provider education to ensure all high-risk individuals receive appropriate screening recommendations. Discrepancies between our findings and prior research may stem from differences in sample size. Larger studies may have greater power to detect significant associations compared to our study of 828 respondents. Additionally, our cohort included only LCS-eligible patients with CLD, whereas other studies examined all at-risk individuals eligible for LCS.

Despite the Centers for Medicare & Medicaid Services (CMS) mandate for shared decision-making in LCS counseling [5], our results suggest inconsistent implementation, likely due to both patient- and provider-related factors. A 2023 survey found that approximately 25% of American adults miss regular checkups, reducing opportunities for screening discussions, including LCS [34]. Patient barriers to LCS include unfamiliarity with screening practices, concerns about false positives, lack of culturally appropriate information, insurance coverage uncertainty, and emotional distress [34,35]. Shared decision-making as a modality in LCS discussion aims to address these knowledge gaps and improve screening uptake. Provider-level barriers include a lack of familiarity with LCS guidelines, skepticism about its efficacy, and perceived low priority [22,35]. Strategies such as LCS-related webinars, visit-based clinical reminders in electronic medical records, and improved physician understanding of radiological data have been proposed to address these barriers [35,36].

This study has several limitations. First, reliance on self-reported data introduces potential recall bias, affecting the accuracy of responses regarding LCS discussions. Second, the cross-sectional study design limits causal inferences between provider discussions and increased screening uptake. Additionally, the use of survey-based retrospective data limits our ability to integrate more granular data at the patient level (type of CLD, comorbidities, disease severity, pack-years of smoking, etc.) or at the provider level (knowledge, attitudes, and barriers to discussing LCS), which limits our understanding of how some of these factors impact low discussion rates. Moreover, prior screening discussions (e.g., breast and colorectal cancer) may not necessarily reflect an individual’s willingness to engage with healthcare providers, as some participants may not have previously met the eligibility criteria or had an indication for such screenings. Lastly, our dataset may not fully represent all individuals with CLDs, particularly those who do not regularly engage with the healthcare system, affecting the generalizability of our findings.

## Conclusions

Our study highlights a significant gap in LCS discussions among individuals with CLD, despite their elevated risk. Our findings suggest that prior preventive screening discussions and socioeconomic factors influence LCS discussions. The disparities observed among food-insecure respondents underscore the need for targeted interventions to improve screening uptake. Future efforts should focus on enhancing provider education, integrating shared decision-making, and addressing healthcare disparities through policy and community-based initiatives. By improving the frequency and quality of LCS discussions, we can increase screening adherence and ultimately reduce lung cancer-related morbidity and mortality.
